# Supplementary material for: A Global Screen for Assembly State Changes of the Mitotic Proteome by SEC-SWATH-MS
Source: Cell Syst. 2020 Feb 26;10(2):133–155.e6. doi: 10.1016/j.cels.2020.01.001 (PMC7042714; doi:10.1016/j.cels.2020.01.001)

Q9Y5J1 | UTP18_HUMAN | UTP18 WDR50 CDABP0061 CGI−48
Monomer MW [kDa]: 62.004 Monomer expected elution fraction: 44

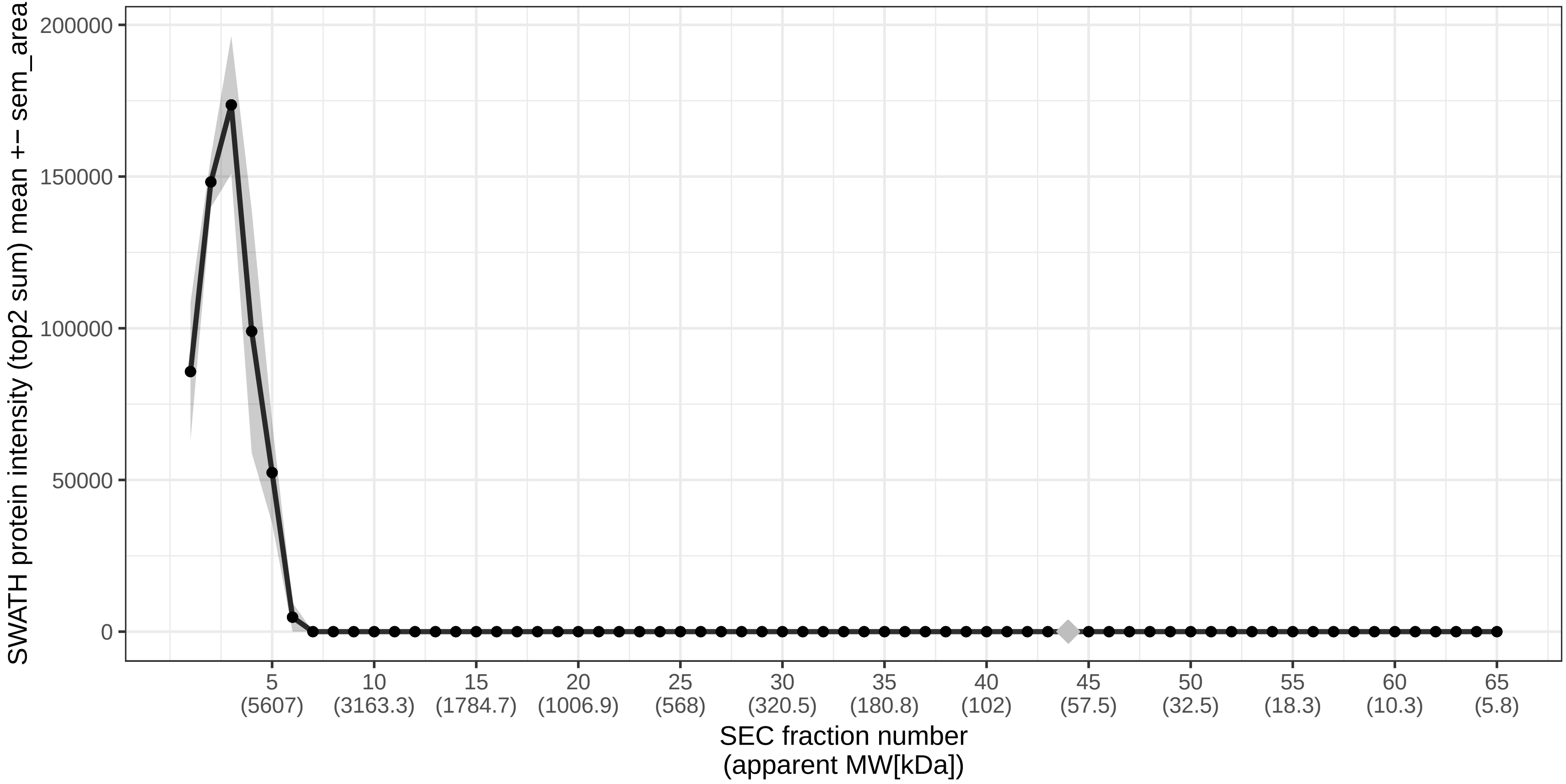

Supplement: Data S1. SEC-SWATH-MS Protein Chromatograms, Related to Figure 1 [file mmc6.zip › SECchrom_Q9Y5J1_UTP18_HUMAN_UTP18_WDR50_CDABP0061_CGI-48.pdf]
